# ﻿Two new species of *Hedyotis* L. (Rubiaceae) from Yunnan and Guangxi, China

**DOI:** 10.3897/phytokeys.263.163043

**Published:** 2025-09-24

**Authors:** Yi-Da Xu, Ming-Deng Yuan, Rui-Jiang Wang

**Affiliations:** 1 State Key Laboratory of Plant Diversity and Specialty Crops, South China Botanical Garden, Chinese Academy of Sciences, Guangzhou, Guangdong 510650, China South China Botanical Garden, Chinese Academy of Sciences Guangzhou China; 2 Key Laboratory of National Forestry and Grassland Administration on Plant Conservation and Utilization in Southern China, South China Botanical Garden, Chinese Academy of Sciences, Guangzhou, Guangdong 510650, China South China Botanical Garden, Chinese Academy of Sciences Guangzhou China

**Keywords:** *Hedyotis-Oldenlandia* complex, phylogeny, pollen, taxonomy

## Abstract

Two new species, *Hedyotis jinghongensis* from Yunnan Province and *H. austroguangxiensis* from the Guangxi Zhuang Autonomous Region, are described and photographed here. *H. jinghongensis* is similar to *H. communis* and *H. interrupta* in having lanceolate to narrowly lanceolate leaves and triangular stipules, but it differs from them by its smaller leaves, stipules marginally with 3–6 colleter-tipped linear lobes on each side, and shorter inflorescences. *H. austroguangxiensis* is most similar to *H. taishanensis* and *H. shenzhenensis* in having terminal, compound-cymose inflorescences and short internodes at the base, but it differs from them by its terete stem and inflorescence rachis and longer corolla tubes. Molecular phylogenetic analysis revealed that the two new species represent independent clades. In addition, they were assessed as Least Concern (LC) according to the IUCN Red List Categories and Criteria.

## ﻿Introduction

The *Hedyotis-Oldenlandia* complex is one of the largest and most controversial species groups in the tribe Spermacoceae of Rubiaceae. Due to high morphological diversity, the generic delimitation within this complex has long been debated (Guo et al. 2013). In some studies, the complex was merged into a broadly circumscribed *Hedyotis* L. (1753: 101), based on shared morphological characters such as herbaceous or shrubby habits, relatively small, mostly 4-merous flowers, and bilocular capsular fruits containing a few to many small seeds (Lamarck 1792; Willdenow 1798; Roemer and Schultes 1818; Steudel 1821; Dutta and Deb 2004). However, phylogenetic studies have demonstrated that this complex is polyphyletic and should be subdivided into several genera (Groeninckx et al. 2009; Guo et al. 2013; Wikström et al. 2013; Neupane et al. 2015; Gibbons 2020). As currently defined, the genus *Hedyotis* s. str. is characterized by erect and robust herbs or shrubs with entire stipules that have colleter-tipped serrate margins and diplophragmous capsules (Guo et al. 2013). It comprises approximately 150 species distributed mainly in the Asia-Pacific biogeographic region, where the modern distribution center of this genus occurs.

While examining specimens of *Hedyotis* s. str. collected from China at the herbaria of IBSC, KUN, HITBC, IBK, GXMG, GXMI, and PE, we found two misidentified species. Further field surveys, morphological comparisons with similar species from China and adjacent countries, and molecular phylogenetic analyses indicated that these two species are new to science and are therefore formally described here.

## ﻿Materials and methods

Morphological data were collected from living individuals and specimens deposited at the herbarium of the South China Botanical Garden, Chinese Academy of Sciences (IBSC). Pollen and seeds were observed using a scanning electron microscope (SEM, JSM-6360LV) at an accelerating voltage of 15.00 kV. Pollen terminology followed Punt et al. (2007).

The conservation assessment was undertaken according to the guidelines for assessing the conservation status of species (IUCN 2024). Estimation of the extent of occurrence (EOO) and area of occupancy (AOO) was performed in GeoCAT (Bachman et al. 2011) using 2 × 2 km grid cells.

A total of 32 samples representing 29 taxa were included in the phylogenetic study (Table 1). Of these, *Agathisanthemum bojeri*Klotzsch (1861: 294) and *Pentodon pentandrus*Vatke (1875: 231) were selected as outgroups. Five DNA markers, namely the nuclear internal transcribed spacer (ITS) region and the chloroplast petD, rps16, trnH–psbA, and trnL–F regions, were employed to reconstruct the phylogenetic tree. Total genomic DNA was extracted from silica-dried leaves. DNA extraction and PCR methods followed Guo et al. (2011). The sequences of other taxa included in the analysis were downloaded from GenBank (Table 1). Sequence alignment was first performed using MUSCLE v. 3.8.31 (Edgar 2004) with default settings and then manually edited using BioEdit (Hall 1999). Maximum likelihood (ML) analysis was implemented in IQ-TREE v. 2.0 (Nguyen et al. 2015) using the TIM+F+R2 substitution model selected by ModelFinder (Kalyaanamoorthy et al. 2017). Bayesian inference (BI) analysis was conducted using MrBayes v. 3.2.7 (Ronquist et al. 2012), with GTR+G+I as the best-fit nucleotide substitution model selected by MrModeltest v. 2.3 (Nylander 2004). The Markov chain Monte Carlo (MCMC) algorithm was run for 1,000,000 generations with four incrementally heated chains starting from random trees, and one tree was sampled per 100 generations. The initial 25% of the sampled trees were discarded as burn-in, and a 50% majority-rule consensus tree was calculated from the remaining trees, with nodal support summarized as posterior probabilities (PP).

**Table 1.** The taxa, vouchers, and GenBank accession numbers of nrITS, petD, rps16, trnH-psbA, and trnL-F sequences for phylogenetic analysis.

Taxon	Voucher (herbarium)	nrITS	petD	rps16	trnH-psbA	trnL-F
*Agathisanthemum bojeri* Klotzsch	Dessein et al. 671 (BR)	AM939424	EU557678	EU543018	/	EU543077
*Hedyotis acutangula* Champ. ex Benth.	Rui-Jiang Wang HA_02 (IBSC)	JX111197	JX111085	JX111241	JX111160	JX111316
*Hedyotis cantoniensis* F.C.How ex W.C.Ko	Rui-Jiang Wang *et al*. 1250 (IBSC)	JF699912	JF700061	JX111247	JF699773	JX111322
*Hedyotis cathayana* W.C.Ko	Guo-Bin Jiang 1071 (IBSC)	MZ325999*	MZ468103*	MZ343044*	MZ514105*	MZ514115*
*Hedyotis caudatifolia* Merr. & F.P.Metcalf	Xing Guo et al. 1269 (IBSC)	JF699916	JF700065	JX111256	JF699777	JX111329
*Hedyotis communis* W.C.Ko	Bo Li LB0171 (IBSC)	JX111208	JX111094	JX111257	JX111167	JX111330
*Hedyotis consanguinea* Hance	Rui-Jiang Wang 1254 (IBSC)	JF699923	JF700071	JX111258	JF699783	JX111331
*Hedyotis cryptantha* Dunn	Rui-Jiang Wang et al. 1152 (IBSC)	JX111209	JX111095	JX111260	JX111168	JX111333
*Hedyotis effusa* Hance	Rui-Jiang Wang et al. 1268_1 (IBSC)	JF699933	JF700083	JX111262	JF699790	JX111335
*Hedyotis exserta* Merr.	Guo-Bin Jiang & Xin-Xin Zhou 1124 (IBSC)	MT345066	MT347606	MT792387	MT792403	MZ514116
*Hedyotis interrupta* G.B.Jiang & R.J.Wang	Guo-Bin Jiang et al. 1136_2 (IBSC)	MT345072	MT347612	MT792393	MT792409	MZ514117*
*Hedyotis jinghongensis* M.D.Yuan & R.J.Wang	Ming-Deng Yuan et al. YS26 (IBSC)	MW166245*	MW165874*	MW405421*	MZ514109*	MZ514122*
*Hedyotis jinghongensis* M.D.Yuan & R.J.Wang	Ming-Deng Yuan et al. 1406 (IBSC)	MZ326002*	/	MZ343049*	MZ514106*	MZ514119*
*Hedyotis jinghongensis* M.D.Yuan & R.J.Wang	Xin-Xin Zhou et al. 5469 (IBSC)	MZ326009*	MZ468112*	MZ343056*	MZ514107*	MZ514120*
*Hedyotis loganioides* Benth.	Rui-Jiang Wang 1247 (IBSC)	JF699909	JF700058	JX111245	JF699770	JX111319
*Hedyotis longiexserta* Merr. & F.P.Metcalf	Ming-Deng Yuan et al. YS60 (IBSC)	MW396581*	MW405435*	MW405424*	/	MZ514123*
*Hedyotis matthewii* Dunn	Ming-Deng Yuan et al. YS236 (IBSC)	MW396582*	MW405438*	MW405427*	MZ514111*	MZ514125*
*Hedyotis nankunshanensis* R.J.Wang & S.J.Deng	Rui-Jiang Wang et al. 1688 (IBSC)	JN975969	JN975964	OQ723460	OQ723461	OQ723462
*Hedyotis nanlingensis* R.J.Wang	Ming-Deng Yuan et al. YS228 (IBSC)	MW396579*	MW405437*	MW405426*	MZ514110*	MZ514124*
*Hedyotis austroguangxiensis* M.D.Yuan & R.J.Wang	Ming-Deng Yuan et al. YS340(IBSC)	MZ326011*	MZ468122*	MZ343065*	MZ514112*	MZ514126*
*Hedyotis austroguangxiensis* M.D.Yuan & R.J.Wang	Ming-Deng Yuan & Yi-Da Xu YS430 (IBSC)	MZ326013*	MZ468125*	/	MZ514113*	MZ514127*
*Hedyotis paridifolia* Dunn	Rui-Jiang Wang et al. 1162 (IBSC)	JX111220	JX111106	JX111272	JX111179	JX111346
*Hedyotis prostrata* Blume	Dan-Liang, Guo-Bin Jiang et al. 1187 (IBSC)	MT345074	MT347614	MT792396	MT792412	MZ514118
*Hedyotis pubirachis* Y.D.Xu & R.J.Wang	Dan Liang et al. WP1352 (IBSC)	MW264178	MW266053	MZ447122	MZ447123	MZ447125
*Hedyotis shenzhenensis* T.Chen	Rui-Jiang Wang et al. 1262_1 (IBSC)	JF699951	JF700101	JX111276	JF699805	JX111350
*Hedyotis taishanensis* G.T.Wang & R.J.Wang	Dan Liang et al. WP1330 (IBSC)	MZ479676*	MZ514102*	MZ514103*	MZ514108*	MZ514121*
*Hedyotis tenuipes* Hemsl. ex F.B.Forbes & Hemsl.	Rui-Jiang Wang 1234_1 (IBSC)	JF699960	JF700110	JX111280	JF699812	JX111354
*Hedyotis uncinella* Hook. & Arn.	Rui-Jiang Wang 1217 (IBSC)	JF699963	JF700113	JX111282	JF699814	JX111356
*Hedyotis vachellii* Hook. & Arn.	Unknown YU21 (CUHK)	HQ148823	/	HM752981	HM640381	HM752896
*Hedyotis xinyiensis* X.Guo & R.J.Wang	Rui-Jiang Wang 1182 (IBSC)	JF699970	JF700120	JX111288	JF699820	JX111362
*Hedyotis yangchunensis* W.C.Ko & Zhang	Rui-Jiang Wang 1270-1 (IBSC)	JF699972	JF700122	JX111290	JF699821	JX111364
*Pentodon pentandrus* Vatke	Dessein et al. 598 (BR)	AM939528	EU557759	AF003612	/	EU543154

Notes: '*' indicates that the sequences are newly added, and the missing sequences are marked with ‘/’.

## ﻿Results

### ﻿Phylogenetic analysis

**Figure 1.**
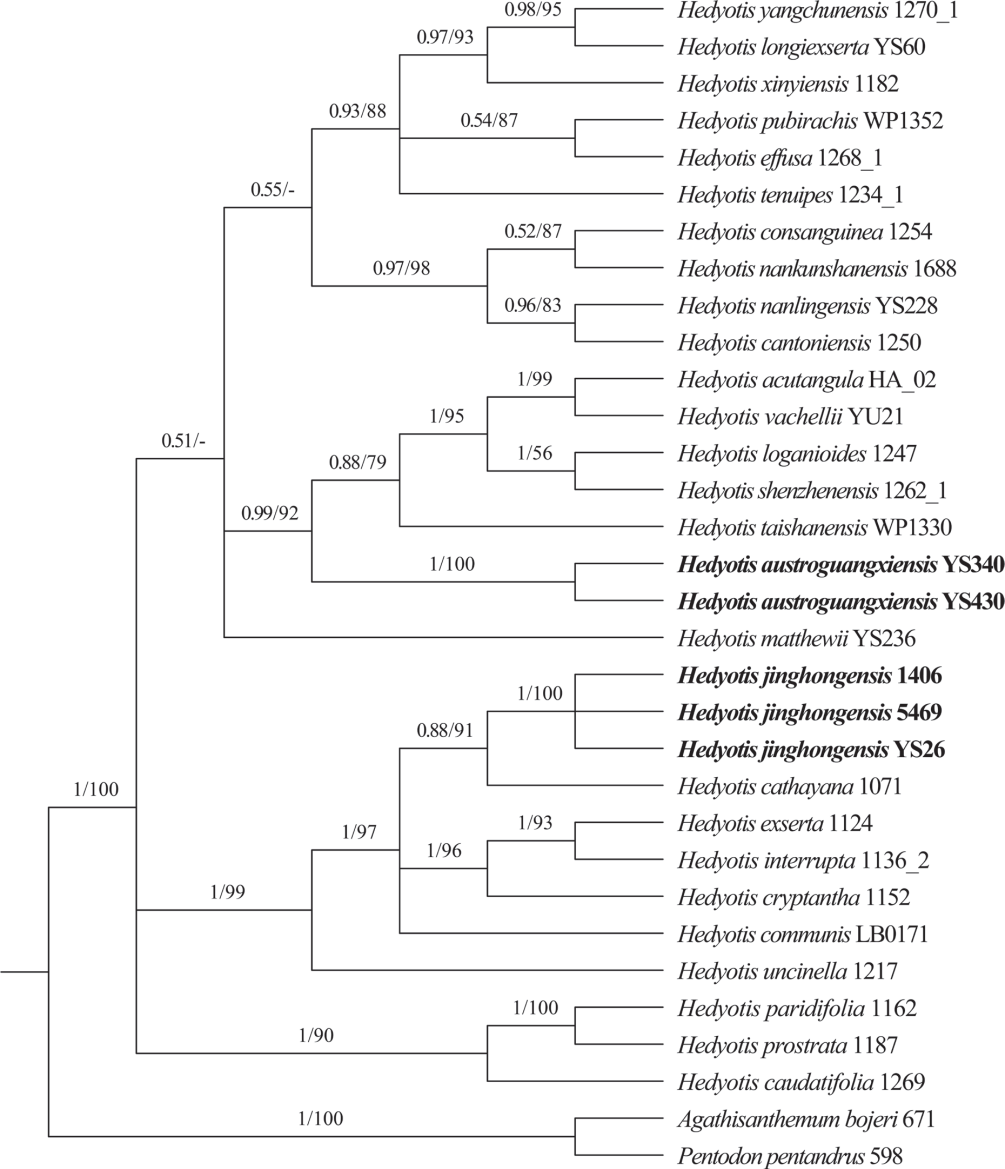
Phylogenetic relationships of *Hedyotis* s. str. based on a combined analysis of nuclear ITS and four chloroplast regions (petD, rps16, trnH–psbA, and trnL–F). Bayesian posterior probabilities (PP ≥ 0.50) and bootstrap values (BS ≥ 50%) are indicated above branches (PP left, BS right).

The phylogenetic analysis based on combined nuclear ITS and four plastid markers (petD, rps16, trnH–psbA, and trnL–F) resolved all the samples of the two novel species within the core *Hedyotis* clade, forming two independent clades. *H. austroguangxiensis* is sister to the lineage of (((*H. vachellii* + *H. acutangula*) + (*H. loganioides* + *H. shenzhenensis*)) + *H. taishanensis*) (PP = 0.99, BS = 92). *H. jinghongensis* is sister to *H. cathayana* (PP = 0.88, BS = 91; Fig. 1).

### ﻿Taxonomic treatment

#### ﻿1. Hedyotis jinghongensis

Taxon classificationPlantaeGentianalesRubiaceae

M.D.Yuan & R.J.Wang sp. nov.

5616C4CD-FAFD-5A2A-956B-2163E809C8AD

urn:lsid:ipni.org:names:77369658-1

Figs 2, 3

##### Type.

CHINA • From Yunnan Province: Dai Autonomous Prefecture of Xishuangbanna, Jinghong City, Jinuo Town, roadside, under thick forest, 21°58'50.29"N, 101°04'21.16"E, ca. 1288 m a.s.l., flowering, short-styled flower, 31 July 2019, *Xin-Xin Zhou, Guo-Bin Jiang & Ming-Deng Yuan 1406* (holotype: IBSC [0858378]; isotype IBSC [0858377]).

**Figure 2.**
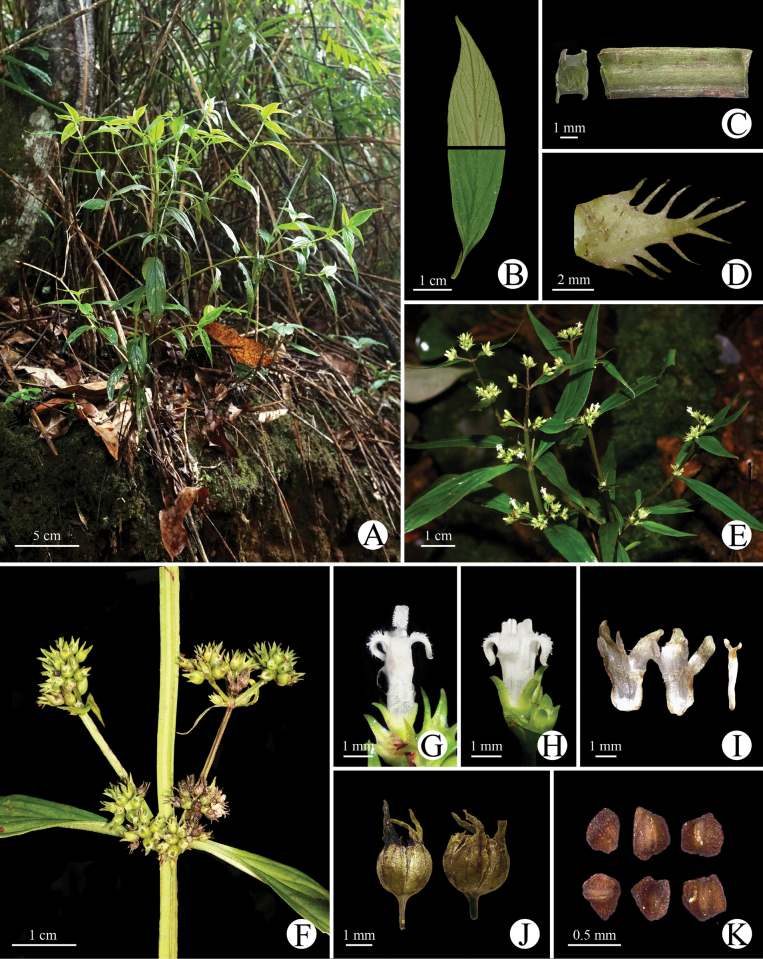
*Hedyotis jinghongensis*. (A–D, H, I from Ming-Deng Yuan et al. 1406; E, G from Yan-Xiong Gong G38; F, J, K from Xin-Xin Zhou 5469). A. Habit; B. Abaxial (above) and adaxial (below) sides of leaf blade; C. Tetragonal stem (right) and transverse section (left); D. Stipule; E. Individual; F. Infructescences; G. Long-styled flower; H. Short-styled flower; I. Longitudinal section of short-styled flower; J. Dehisced capsules; K. Seeds.

##### Diagnosis.

The new species is similar to *H. communis* W.C.Ko (1974: 578) and *H. interrupta* G.B.Jiang & R.J.Wang (2021) in having lanceolate to narrowly lanceolate leaves and triangular stipules, but it differs from *H. communis* by having obvious tetragonal stem, smaller leaves, stipules marginally with 3–6 colleter-tipped linear lobes on each side, and short inflorescences with small flowers (Table 2). It is also distinguished from *H. interrupta* by having small leaves, stipules marginally with 3–6 colleter-tipped linear lobes on each side, and short inflorescences (Table 2).

**Table 2.** Morphological comparison among *Hedyotis jinghongensis*, *H. communis*, and *H. interrupta*.

Characters	*H. jinghongensis*	*H. communis*	*H. interrupta*
Habit	herbs, 15–60 (–80) cm tall	subshrubs or herbs, to 1.5 m tall	subshrubs or herbs, to 1.5 m tall
Stem	tetragonal, narrowly winged	terete	tetragonal, winged
Petiole length (mm)	2.0–5.0	subsessile	1.5–4.0
Leaf size (cm)	2.5–9.0 × 0.7–2.0	10–15 × 1.5–2.0	9.0–17.0 × 2.0–5.0
Leaf shape	lanceolate to narrowly lanceolate	narrowly elliptic-oblong or elliptic	oblong to lanceolate
Stipule	triangular to narrowly triangular, marginally with 3–6 colleter-tipped linear lobes on each side	triangular to narrowly triangular, setose at margin	broadly triangular, marginally with 8–10 colleter-tipped teeth
Inflorescence	terminal and axillary on lateral branches, cyme or compound cymose	axillary, 7.0–24 cm long, compound-cymose to paniculate	axillary, 3.0–10.0 cm long, compound-cymose to paniculate
Length of Corolla tube (mm)	1.7–2.0	4.5–5.0	5.0–6.0
Capsules	subglobose, 1.5–2.0 × 2.0–2.5 mm	obovoid or subglobose, 2.0–3.0 mm in diameter	ellipsoid to subglobose, 1.5–2.0 mm in diameter

##### Description.

Perennial herbs, erect, 15–60 (–80) cm tall. ***Stem*** tetragonal, narrowly winged, branched mostly at the upper part, glabrous. ***Leaves*** opposite, petioles 2.0–5.0 mm long, glabrous; blade 2.5–9.0 × 0.7–2.0 cm, lanceolate to narrowly lanceolate, apex acuminate to caudate acuminate, base cuneate, blade papyraceous, light green adaxially and grayish green abaxially, glabrous on both sides; midrib depressed adaxially and prominent abaxially, secondary veins 3–7 on each side, distinct on both sides. ***Stipules*** interpetiolar, 5.0–10 mm long, triangular to narrowly triangular, marginally with 3–6 colleter-tipped linear lobes on each side, glabrous. ***Inflorescences*** terminal and axillary on lateral branches, 5–20 flowered, cyme or compound cymose; peduncles 0.5–3.0 cm long, tetragonal, narrowly winged, glabrous; bracts 2.0–12.0 mm long, lanceolate or linear. ***Flowers*** heterostylous, pedicels 0–2.0 mm long. ***Hypanthium*** ca. 1.0 mm long, campanulate, glabrous; sepals 4, 1.0–1.2 mm long, narrowly triangular, glabrous. ***Corolla*** tubular, white; tube 1.7–2.0 mm long, densely pubescent at throat adaxially, glabrous abaxially; lobes 4, 1.8–2.0 mm long, oblong-lanceolate, glabrous abaxially, and pubescence adaxially. Stigma bilobed, ca. 0.5 mm long, clavate. Ovary 2-celled, ovules numerous on axile placentas. Stamens 4, anthers ca. 0.8 mm long, oblong-linear. ***Long-styled flowers***: corolla tube ca. 2.0 mm long; stamens included, adnate to the middle of corolla tube; filaments ca. 0.8 mm long; styles ca. 3.3 mm long, stigma exserted. ***Short-styled flowers***: corolla tube ca. 1.7 mm long; stamens included, adnate to throat of corolla tube; filaments ca. 1.2 mm long, anthers exserted; styles ca. 2.8 mm long, stigma included or slightly exserted. ***Fruits*** capsular, 1.5–2.0 × 2.0–2.5 mm, subglobose, sepals persistent, glabrous, dehiscent at apex and then septicidally along the ventral suture. ***Seeds*** ca. 0.5 mm long, numerous, angular, 0.4–0.6 mm long, testa black, reticulated.

##### Phenology.

Flowering and fruiting individuals were observed between June and December.

##### Etymology.

The specific epithet refers to the type locality of this new species, Jinghong City, Dai Autonomous Prefecture of Xishuangbanna, Yunnan Province. Its Chinese name is “景洪耳草” (Jǐng Hóng Ěr Cǎo).

##### Distribution and habitat.

Currently, *Hedyotis jinghongensis* is only known from southern Yunnan Province, China. It grows on roadsides under secondary forests at an elevation of 700–1400 m. The main associated species are *Selaginella leptophylla* Baker (Selaginellaceae), *Phyllanthus urinaria* Linnaeus (Phyllanthaceae), and *Sonerila cantonensis* Stapf (Melastomataceae).

##### Palynology.

**Figure 3.**
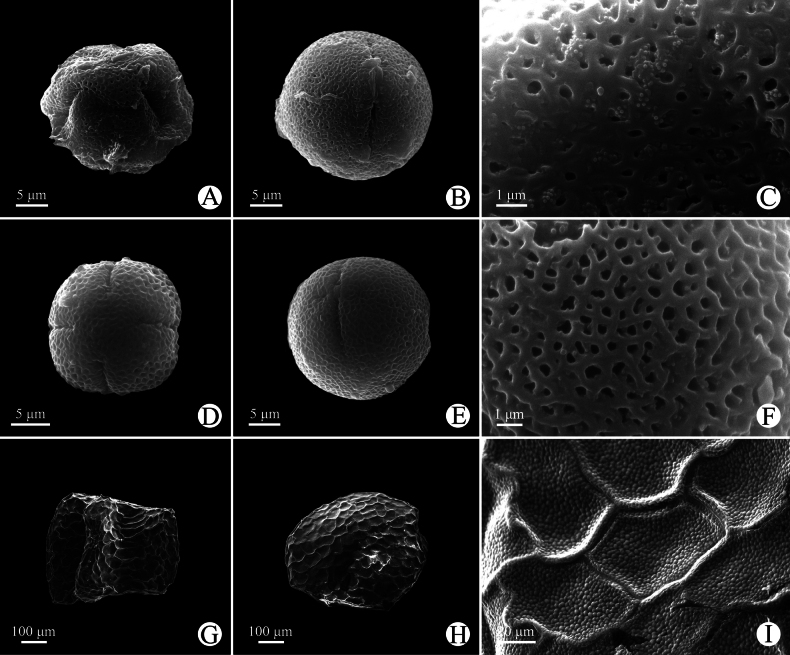
Micromorphology of pollen and seed of *Hedyotis jinghongensis* under SEM. (A–C pollen grains from Yan-Xiong Gong G38, long-styled flower; D–F pollen grains from Ming-Deng Yuan et al. 1406, short-styled flower; G–I seeds from Xin-Xin Zhou 5469). A, D. Polar view; B, E. Equatorial view; C, F. Reticulate ornamentation of pollen grains; G–I. Ventral side, dorsal side, and ornamentation of seeds, respectively.

The pollens of the new species are monads, isopolar, with 3- or 4-colporate apertures, the tectum is double microreticulate, with a psilate suprareticulum and a microechinate infrareticulum. The pollen size is 25.9 (23.6–28.0) × 24.8 (23.4–26.3) μm with P/E value 1.04, and the shape is spheroidal in long-styled flowers (Fig. 3A–C); the pollen size is 20.0 (17.5–20.2) × 16.9 (16.4–17.1) μm with P/E value 1.18, and the shape is subprolate in short-styled flowers (Fig. 3D–F).

##### Additional specimens examined.

**CHINA** • Yunnan: Dai Autonomous Prefecture of Xishuangbanna, Cheli County, Zhalei, under thickets, 1400 m a.s.l., Oct 1936, *Qi-Wu, Wang 79170* (KUN); • Jinghong City, Jinuo Town, Longpa Village, under forest, 22°2'N, 101°1'E, 1250 m a.s.l., 02 Aug 1977, *Guo-Da Tao 16483* (HITBC, IBSC); • *ibid.*, 04 Aug 1977, *Guo-Da Tao 17845* (HITBC); • *ibid.*, 21°59'N, 101°40'E, 1250 m a.s.l., 29 Jul 1977, *Guo-Da Tao 17850* (HITBC, KUN); • *ibid.*, 30 Nov. 2018, *Xin-Xin Zhou 5469* (IBSC); • *ibid.*, Mengyang Town, near river, 22°53'N, 100°53'E, 750 m a.s.l., *Guo-Da Tao 16799* (HITBC); • *ibid.*, Guanping Village, roadside, slopes, 22°11'N, 100°52'E, 800 m a.s.l., 17 Aug 1977, *Guo-Da Tao 17577* (KUN, IBSC); • *ibid.*, 10 Nov 2019, *Ming-Deng Yuan & Jiang-Ping Shu YS26* (IBSC); • *ibid.*, Puwen Town, Dakaihe Village, under forest, 22°35'N, 101°2'E, 14 Aug 1977, *Guo-Da Tao 16898* (HITBC, KUN); • *ibid.*, Mengla County, Mengman Town, Jingpiao Village, 21°37'N, 101°41'E, *Investigation Team 25230* (HITBC); • *ibid.*, Yaoqu Town, Nazuo Village, in miscellaneous wood forest, 21°40'N, 101°36'E, 700–800 m a.s.l., 23 Nov 1974, *Zeng-Hong Yang 012484* (HITBC, KUN); • *ibid.*, Puer City, Jinggu County, 23°24'7.35"N, 100°59'50.87"E, 1224 m a.s.l., 12 Sept 2020, *Yan-Xiong Gong G38* (IBSC); • *ibid.*, Puer County, Mohei Town, Choushui Village, 23°16'N, 101°10'E, 1300 m a.s.l., 27 Aug 1990, *Guo-Da Tao 46403* (HITBC); • *ibid.*, 09 Nov 2019, *Ming-Deng Yuan & Jiang-Ping Shu YS5* (IBSC); • *ibid.*, the road along Puer County to Jinggu County, in the dank of the valley, 23°21'N, 100°57'E, 950 m a.s.l., *Hong Wang 4805* (HITBC).

##### Conservation status.

So far, four subpopulations with more than 500 mature individuals have been found in the field (AOO 40 km^2^, EOO 8500 km^2^). According to the criteria D1 of the IUCN Red List Categories and Criteria (IUCN 2024), the species can be assessed as “Vulnerable”. However, many other subpopulations of this species may be found nearby in southern Yunnan or northern Laos. Considering that this species has no economic uses, we recommend evaluating it as Least Concern (LC).

#### ﻿2. Hedyotis austroguangxiensis

Taxon classificationPlantaeGentianalesRubiaceae

M.D.Yuan & R.J.Wang sp. nov.

309E7C6F-C755-5525-A896-C31DA8ACA26B

urn:lsid:ipni.org:names:77369659-1

Figs 4, 5

##### Type.

CHINA • Guangxi: Chongzuo City, Ningming County, Tongmian Town, roadside, 21.711643, 107.521562, ca. 830 m a.s.l., flowering, long-styled flower, 24 April 2021, *Ming-Deng Yuan & Yi-Da Xu YS430* (holotype: IBSC [0868775]); isotype: IBSC [0868776]).

**Figure 4.**
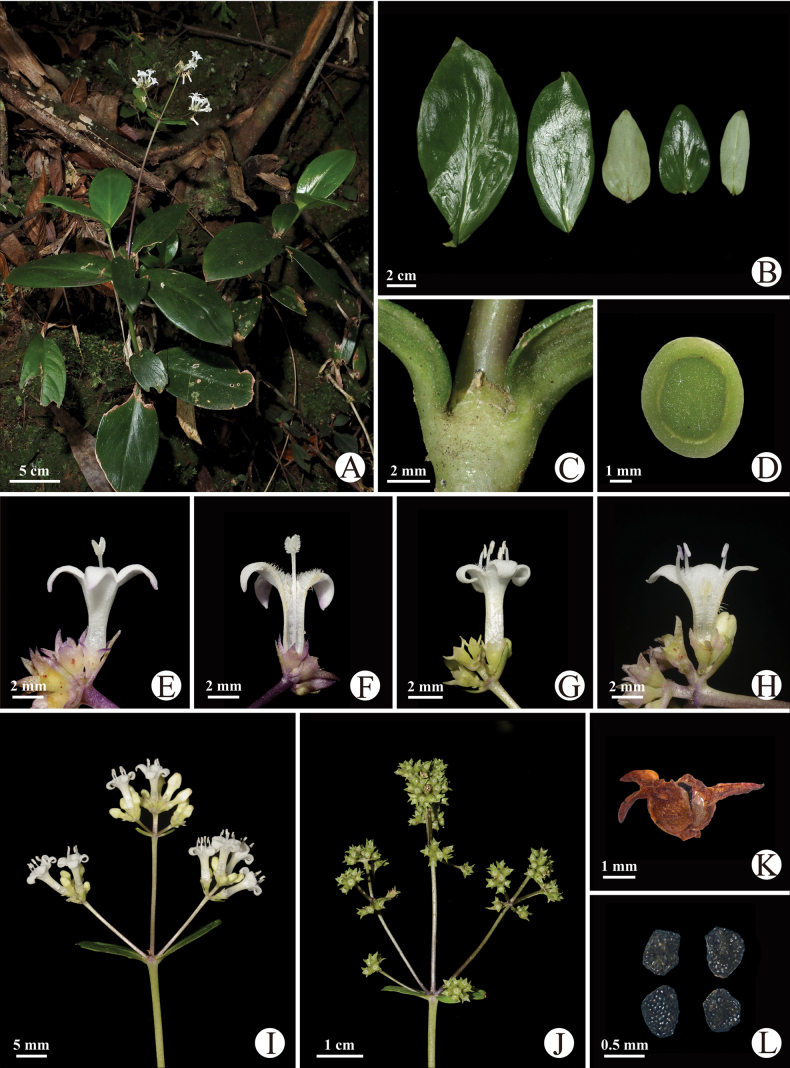
*Hedyotis austroguangxiensis*. (A–D, G–I from Ming-Deng Yuan & Yi-Da Xu YS431; E, F from Ming-Deng Yuan & Yi-Da Xu YS430; J–L from Ming-Deng Yuan et al. YS340). A. Habit; B. Leaf blades; C. Stipule; D. Transverse section of stem; E, F. Long-styled flower; G, H. Short-styled flower; I. Inflorescence; J. Infructescence; K. Dehisced capsules; L. Seeds.

##### Diagnosis.

*Hedyotis austroguangxiensis* is similar to *H. taishanensis* G.T. Wang (Wang et al. 2018) and *H. shenzhenensis* Tao Chen (2007) in having shortened internodes at the base and terminal, compound-cymose inflorescences. But it can be differed from *H. taishanensis* by its terete stem and inflorescence rachis, obovate to ovate or oblong leaves, 3–6 secondary veins on each side, and longer corolla tubes; it differs from *H. shenzhenensis* by its terete stems and inflorescence axes, smaller leaves, and longer corolla tubes (Table 3).

**Table 3.** Morphological comparison among *Hedyotis austroguangxiensis*, *H. taishanensis*, and *H. shenzhenensis*.

Characters	*H. austroguangxiensis*	*H. taishanensis*	*H. shenzhenensis*
Stem	terete	tetragonal	tetragonal
Leaf size (cm)	2.5–15 × 1.5–7.0	6.7–17.9 × 1.9–3.8	8.5–15 × 5.0–9.0
Leaf shape	ovate to oblong	lanceolate to ovate-lanceolate	elliptic, elliptic-oblong, or obovate
Secondary veins	4–6 on each side	6–7 on each side	4–6 on each side
Stipule	broadly triangular, glabrous	broadly triangular, pubescent	triangular, pubescent
Inflorescence rachis	terete	tetragonal and sulcate	tetragonal and sulcate
Length of Corolla tube (mm)	4.2–5.2	2.0–3.2	ca. 3.0

##### Description.

Perennial herbs, erect, 20–50 cm tall. ***Stem*** terete, glabrous, internodes 0.5–5.0 cm long at the base and 5.0–13.0 cm long toward top. ***Leaves*** opposite, sessile, blade 2.5–15.0 × 1.5–7.0 cm, ovate to oblong, apex acuminate or obtuse, base broadly cuneate to rounded, blade coriaceous, dark green adaxially and grayish green abaxially, glabrous on both sides; midrib concave adaxially and prominent abaxially; secondary veins 4–6 on each side, inconspicuous on both sides. ***Stipules*** interpetiolar, 1.5–2.5 mm long, broadly ovate or triangular, apex acuminate, margin toothed with tipped colleters, glabrous or sometimes fimbriate. ***Inflorescences*** terminal, compound-cymose; rachis terete; peduncles 6.0–15.0 cm long; bracts ovate-lanceolate to lanceolate, 0.5–3.0 cm long; bracteoles lanceolate, 1.0–1.5 mm long. ***Flowers*** heterostylous, subsessile, pedicels 0–0.5 mm long. ***Hypanthium*** ca. 1.0 mm long, obconic-campanulate, glabrous; sepals 4, ca. 1 mm long, triangular, glabrous. ***Corolla*** white or purplish on the margin of corolla lobes, infundibuliform, tube 4.2–5.2 mm long, glabrous abaxially, densely pubescent at throat; lobes 4, ca. 3.0 mm long, ovate. Stigma bilobed, ca. 1.0 mm, clavate. Ovary 2-celled, ovules numerous on axile placentas. Stamens 4, anthers oblong, ca. 1.0 mm long, oblong-lanceolate. ***Long-styled flower***: stamens included, adnate to the middle part of corolla tube; filaments ca. 1.0 mm long; styles exserted, ca. 6.0 mm long. ***Short-styled flower***: stamens exserted, adnate to throat of corolla tube; filaments ca. 2.0 mm long; styles included, ca. 3.0 mm long. ***Fruits*** capsular, ca. 2.0 mm in diameter, subglobose or oblate globose, dehiscent at apex and then septicidally along the ventral suture. ***Seeds*** 0.5–0.7 mm long, numerous, angular, testa black, reticulate.

##### Phenology.

Flowering occurs from March to June, and fruiting from July to January of the following year.

##### Etymology.

The specific epithet refers to the fact that the species is growing in the south of the Guangxi Zhuang Autonomous Region. Its Chinese name is “桂南耳草” (Guì Nán Ěr Cǎo).

##### Distribution and habitat.

Up to now, *Hedyotis austroguangxiensis* is known from Chongzuo City and Fangchenggang City of the Guangxi Zhuang Autonomous Region; it grows mainly on roadsides or wet rocks under secondary forests at an elevation of 800–1100 m. Main associated species are *Selaginella doederleinii* Hieron. (Selaginellaceae) and *Blechnopsis orientalis* (L.) C.Presl (Blechnaceae).

##### Palynology.

**Figure 5.**
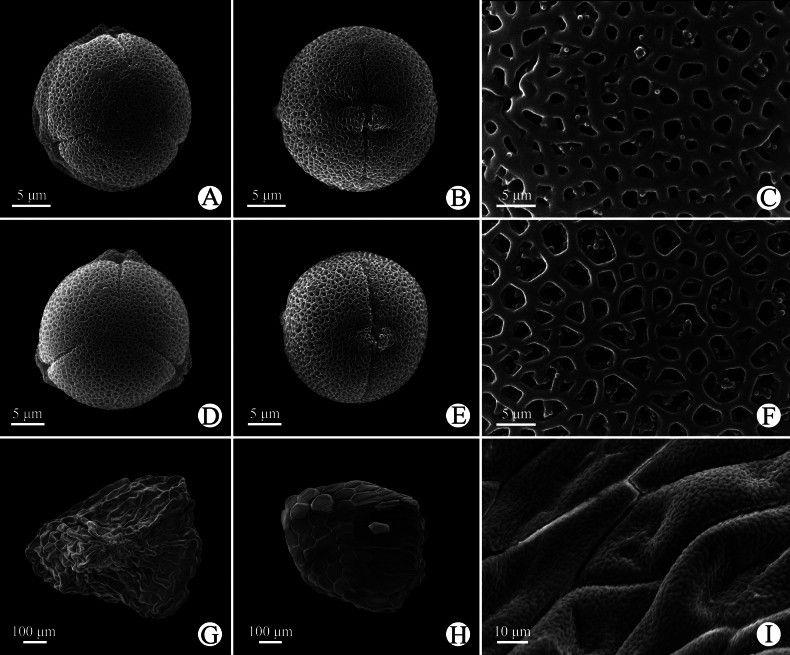
Micromorphology of pollen and seed of *Hedyotis austroguangxiensis* under SEM. (A–C pollen grains from Ming-Deng Yuan & Yi-Da Xu YS430, long-styled flower; D–F pollen grains from Ming-Deng Yuan & Yi-Da Xu YS431, short-styled flower; G–I seeds from Ming-Deng Yuan et al. YS340). A, D. Polar view; B, E. Equatorial view; C, F. Reticulate ornamentation of pollen grains; G–I. Ventral side, dorsal side, and ornamentation of seeds, respectively.

The pollens of the new species are monads, isopolar and spheroidal, with 3-colporate apertures, the tectum is double microreticulate, with a psilate suprareticulum and a microechinate infrareticulum. The pollen size is 21.7 (20.2–22.7) × 20.6 (19.5–21.4) μm with P/E value of 1.05 in long-styled flower (Fig. 5A–C) and 23.3 (21.1–24.3) × 22.5 (21.3–24.1) μm with P/E value of 1.04 in short-styled flower (Fig. 5D–F).

##### Additional specimens examined.

**CHINA** • Guangxi Zhuang Autonomous Region: Chongzuo City, Ningming County, 28 Oct 2020, 21°40'22.476"N, 107°29'09.218"E, 1066.52 m a.s.l., *Ming-Deng Yuan & Yi-Da Xu YS391* (IBSC); • *ibid.*, Aidian Town, Luxu Village, Gongmu Mountain, 14 Mar 2010, *Yu-De Peng, Ying-Feng Huang, Cui-Hong Lu 21786* (GXMG); • *ibid.*, Tongmian Town, Kunan Village, Liangling, 21°42'33.76"N, 107°31'24.23"E, 951 m a.s.l., 28 May 2013, *Dong-Xin Nong, Hai-Feng Ceng, Wei Liu 451422130528052LY* (GXMG); • *ibid.*, 7 Jan 2020, *Ming-Deng Yuan et al. YS99* (IBSC); • *ibid.*, 14 Jun 2020, *Ming-Deng Yuan, Guo-Bin Jiang YS340* (IBSC); • *ibid.*, short-styled flower, 24 Apr 2021, *Ming-Deng Yuan & Yi-Da Xu YS431* (IBSC); • Fangchenggang City, Fangcheng District, under thick forest, on the hill, 23 Mar 2010, *Shiwandashan Collection Team 2189* (IBK).

##### Conservation status.

So far, the subpopulations of *Hedyotis austroguangxiensis* exceed 4 based on the information from specimens and our field expedition, and more than 600 mature individuals have been found in the field (AOO 16 km^2^, EOO 91 km^2^). According to the criteria D1 of the IUCN Red List Categories and Criteria (IUCN 2024), the species can be assessed as “Vulnerable”. However, this species is widely distributed and may also be found in neighboring countries like Vietnam and Laos. In addition, it has not found any medicinal and ornamental values yet. We thus recommend evaluating it as Least Concern (LC).

## ﻿Discussion

*Hedyotis jinghongensis* is morphologically similar to *H. communis* and *H. interrupta*, but it can be distinguished from them by 2.5–9.0 × 0.7–2.0 cm leaves (10–15 × 1.5–2.0 cm in *H. communis* and 9.0–17.0 × 2.0–5.0 cm in *H. interrupta*), stipules marginally with colleter-tipped linear lobes on each side (setose at the margin in *H. communis* and colleter-tipped teeth at the margin in *H. interrupta*), 0.5–3.0 cm long terminal and axillary inflorescences on lateral branches (7.0–24 cm long and axillary in *H. communis*, 3.0–10.0 cm long and axillary in *H. interrupta*), and 1.9–2.3 mm long corolla tubes (4.5–5.0 mm long in *H. communis* and 5.0–6.0 mm long in *H. interrupta*). A detailed comparison among them is provided in Table 2.

*Hedyotis austroguangxiensis* is similar to *H. taishanensis* and *H. shenzhenensis*, but it can be distinguished from the latter two species by its terete stems and inflorescence rachis (vs. tetragonal in both species) and 4.2–5.2 mm long corolla tubes (vs. 2.0–3.2 mm long in *H. taishanensis* and ca. 3.0 mm long in *H. shenzhenensis*). A more detailed comparison is provided in Table 3. In addition, *H. austroguangxiensis* is similar to *H. konhanungensis* B.H. Quang, T.A. Le, K.S. Nguyen & Neupane, a species endemic to Vietnam (Quang et al. 2023). Both species have broadly ovate to triangular stipules and terminal, compound-cymose inflorescences, but they can be distinguished by the color of the abaxial leaf blade (grayish green vs. dark purple to purplish black), petiole length (sessile vs. 4.0–6.0 mm), pedicel length (6.0–15.0 cm vs. 2.0–4.0 cm), sepal shape (triangular vs. ovate or nearly oval), abaxial corolla color (white or purplish on the corolla lobe margins vs. bluish purple), and corolla tube length (4.2–5.2 mm vs. 6.0–7.0 mm).

The phylogenetic analysis shows that *H. jinghongensis* is sister to *H. cathayana* (PP = 0.88, BS = 91), but it can be distinguished from the latter by 2.5–9.0 × 0.7–2.0 cm leaves (vs. 15–25 × 3.0–6.0 cm), stipules with 3–6 colleter-tipped linear lobes on each side (vs. glandular with tipped colleters), and 1.7–2.0 mm long corolla tubes (vs. 6.0–9.0 mm long). *H. austroguangxiensis* is sister to the lineage (((*H. vachellii* + *H. acutangula*) + (*H. loganioides* + *H. shenzhenensis*)) + *H. taishanensis*) (PP = 0.99, BS = 92). Within this lineage, aside from the morphologically similar species *H. taishanensis* and *H. shenzhenensis*, *H. austroguangxiensis* can be easily distinguished from the other three species by its terminal compound-cymose inflorescences (vs. axillary thyrsoid with monochasial sub-axes in *H. acutangula*, axillary cymose or compound cymose in *H. loganioides*, and terminal thyrsoid with monochasial sub-axes in *H. vachellii*).

In addition, we have observed that *H. jinghongensis* is often misidentified as *H. uncinella*Hooker and Arnott (1841: 192) due to their tetragonal stems and short inflorescences. However, *H. jinghongensis* can be clearly distinguished from the latter by its terminal and axillary inflorescences on lateral branches (vs. axillary), lanceolate leaves (vs. ovate to elliptic), glabrous stipules (vs. scabrid), cymose or compound-cymose inflorescences (vs. capitate or glomerulate), and glabrous calyx lobes (vs. ciliate). To better distinguish these two new species from sympatric species, we provide a key that includes all currently known *Hedyotis* s. str. species Yunnan Province and Guangxi Zhuang Autonomous Region.

### ﻿Key to the 11 *Hedyotis* s.s. species distributed to Yunnan Province and Guangxi Zhuang Autonomous Region

**Table:** 

1	Stems terete	**2**
–	Stems tetragonal	**6**
2	Inflorescences axillary, glomerate	***H. platystipula***
–	Inflorescences terminal or axillary in upper leaves, compound-cymose	**3**
3	Corolla tube longer than 4.0 mm	***H. austroguangxiensis***
–	Corolla tube shorter than 4.0 mm	**4**
4	Sepals broadly ovate; inflorescence axes distinctly zigzag-shaped	***H. longiexserta***
–	Sepals triangular; inflorescence axes not zigzag-shaped	**5**
5	inflorescence axes tetragonal, dichasial branching	***H. caudatifolia***
–	inflorescence axes terete, monochasial branching	***H. nanlingensis***
6	Inflorescences compound-cymose	**7**
–	Inflorescences glomerate	**8**
7	Stipules narrowly triangular; flowers homostylous	***H. matthewii***
–	Stipules broadly triangular; flowers heterostylous	***H. zhihaoana***
8	Leaves puberulous on both sides; inflorescences axillary	***H. pubicaulis***
–	Leaves glabrous on both sides; inflorescences terminal and axillary in upper leaves	**9**
9	Leaves lanceolate to narrowly lanceolate; stipule narrowly triangular	***H. jinghongensis***
–	Leaves elliptic, ovate, or ovate-oblong; stipules triangular to broadly triangular	**10**
10	Corolla white or pink; hypanthium scabrid; sepals ca. 1/2 as long as the corolla tube	***H. linearifolia***
–	Corolla white or purple; hypanthium glabrous or puberulous; sepals nearly as long as the corolla tube	***H. cephalophora***

## Supplementary Material

XML Treatment for Hedyotis jinghongensis

XML Treatment for Hedyotis austroguangxiensis

## ﻿Additional information

### Use of AI

No use of AI was reported.

### Author ORCIDs

Yi-Da Xu https://orcid.org/0000-0001-9812-4770

Ming-Deng Yuan https://orcid.org/0000-0001-5730-496X

Rui-Jiang Wang https://orcid.org/0000-0002-4985-8281

### Data availability

All of the data that support the findings of this study are available in the main text.
